# Local sleep-like events during wakefulness and their relationship to decreased alertness in astronauts on ISS

**DOI:** 10.1038/s41526-019-0069-0

**Published:** 2019-05-02

**Authors:** Gaetan Petit, Ana Maria Cebolla, Sara Fattinger, Mathieu Petieau, Leopold Summerer, Guy Cheron, Reto Huber

**Affiliations:** 1 Advanced Concepts Team, European Space Agency, ESTEC, 2200 AG Noordwijk, The Netherlands; 2 Child Development Center, University Children’s Hospital Zurich, 8032 Zurich, Switzerland; 3 Laboratory of Neurophysiology and Movement Biomechanics, ULB Neuroscience Institute, Brussels, Université libre de Bruxelles, 1070 Brussels, Belgium; 4 Department of Child and Adolescent Psychiatry and Psychotherapy, Psychiatric Hospital, University of Zurich, Zurich, Switzerland

**Keywords:** Neuroscience, Databases

## Abstract

Adequate sleep quantity and quality is required to maintain vigilance, cognitive and learning processes. A decrease of sleep quantity preflight and on the International Space Station (ISS) has been reported. Recent counter-measures have been implemented to better regulate sleep opportunities on ISS. In our study, astronauts were allocated enough time for sleep the night before the recordings. However, for proper sleep recovery, the quality of sleep is also critical. Unfortunately, data on sleep quality have yet to be acquired from the ISS. Here, we investigate sleep pressure markers during wakefulness in five astronauts throughout their 6-month space mission by the mean of electroencephalographic recordings. We show a global increase of theta oscillations (5–7 Hz) on the ISS compared to on Earth before the mission. We also show that local sleep-like events, another marker of sleep pressure, are more global in space (*p* < 0.001). By analysing the performances of the astronauts during a docking simulation, we found that local sleep-like events are more global when reaction times are slower (*R*^2^ = 0.03, *p* = 0.006) and there is an increase of reaction times above 244 ms after 2 months in space (*p* = 0.012). Our analyses provide first evidence for increased sleep pressure in space and raise awareness on possible impacts on visuomotor performances in space.

## Introduction

Sleep is regulated by two oscillatory processes: the circadian process and the homoeostatic process. The circadian process oscillates on a near 24 h rhythm and defines the sleep inclination periods.^1^ Under normal conditions, the circadian process is synchronised with the light/dark cycle. Astronauts witness short sunrises 16 times a day, as the International Space Station (ISS) orbits Earth every 90 min. As a countermeasure, an artificial 24 h sleep/wake routine is established to align astronauts’ circadian rhythms with coordinated universal time (UTC). Despite this countermeasure, it has been reported that a majority of the ISS crew members are using sleep promoting medication in response to a sleep deficiency, before and during their mission.^2^

Even though astronauts were allocated enough time to sleep in space, sleep quality might not be sufficient to fully dissipate sleep debt. In space, sleep quality might be impacted by external factors such as microgravity, confinement, circadian misalignment, chronic stress, temperature, light and noise disturbances.^3,4^ So far, experiments have shown sensorimotor and neurovestibular adaptation to the space environment.^5–7^ Yet, no conclusion on changes in the underlying mechanisms regulating sleep in space could be made. Most related ISS studies focus on sleep quantity and chronobiology,^8,9^ because during human spaceflight all-night sleep recording is hard to achieve. Only a few reports from Space Shuttles and the early days of human space stations feature sleep electroencephalographic (EEG) data.^10–12^

Regulated separately but operating in parallel with the circadian process, the homoeostatic process, represented by sleep pressure (i.e., sleep need), builds up with the time spent awake. Scalp EEG measurement allows real-time recordings of cortical activity and is currently the gold standard for sleep quality analysis. EEG markers of high sleep pressure can be observed during wakefulness, by increased theta activity (5–7 Hz) and while asleep by an increase of slow wave activity (SWA) (0.5–4 Hz).^13–15^ Although, theta activity increases with the time awake, a strong circadian modulation can be observed.^16–18^ Dynamic changes in SWA’s topographical distribution and a frontal increase in the number of high amplitude slow waves, have been observed after chronic sleep restriction.^19^ Moreover, the local regulation of SWA seems to be closely related to the capacity for neuroplastic changes.^20^ Recently, it has been shown that similar local sleep-like events can be observed in awake rodents.^21^ Using high-density scalp EEG, local sleep-like events have also been studied in awake humans alongside the increase of sleep pressure^22^ and after a period of sleep deprivation.^23,24^

Here, we analyse how astronauts’ local sleep-like events during wakefulness are impacted throughout their 6-month space mission and we investigate if they are related to visuomotor performances. This analysis is based on high-density wake EEG data collected between 2011 and 2013 on the ISS, as part of the Neurospat experiment.^25^

## Results

### Despite similar sleep and wake quantity, theta power changes from Earth to space recordings

**Fig. 1:**
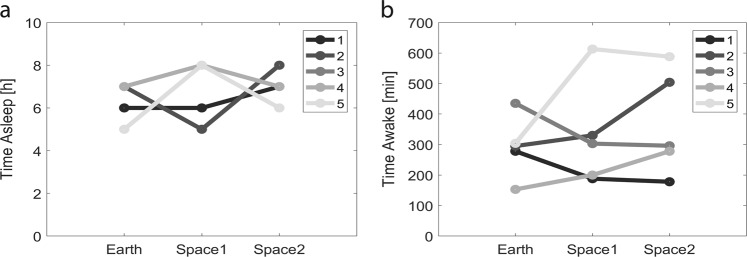
Three conditions: one on Earth, two in Space. **a** For each astronaut, the quantity of sleep the night before the recording is not different across conditions. **b** For each astronaut, the time they have been awake before the beginning of the recording is not different across conditions. (*n* = 5 astronauts)

**Fig. 2:**
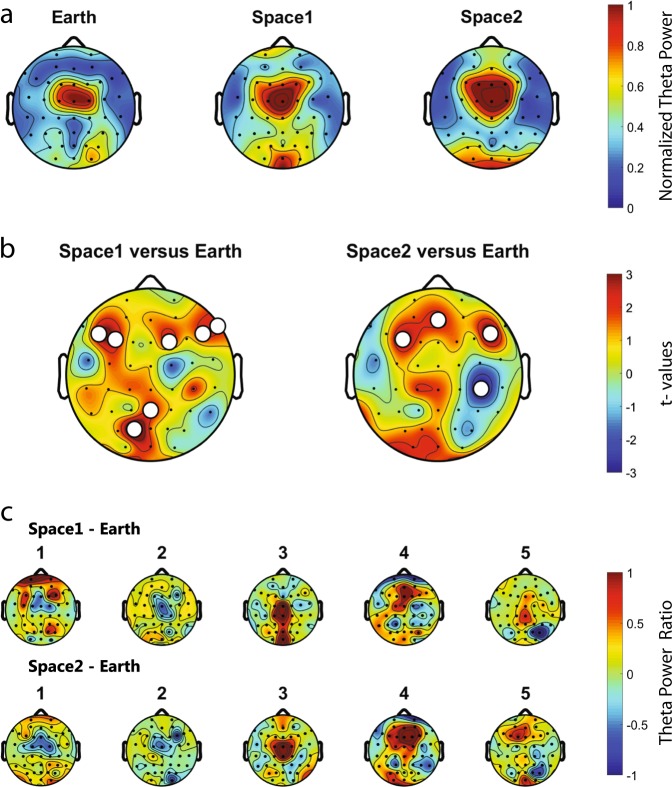
Theta power topographical distribution across the recording conditions. **a** Normalised theta power for Earth, space1 and space2. Consistent central distribution of the theta power in all conditions. **b** In space1 and space2 compared to Earth, theta is globally increased for a majority of astronauts. Red colour indicates an increase of theta power in space compared to Earth (paired *t*-test, *t*-values, white dots uncorrected *p*-values < 0.05). **c** Space1-Earth and space2-Earth differences in theta power for each astronaut (1–5). Differences in theta power from space to Earth are observed but no clear topographical pattern emerges. (*n* = 5 astronauts)

All astronauts were recorded continuously for 70 min while repeatedly docking a simulated Soyuz vehicle to the ISS for the last  25 min of the experiment, this in three conditions: on Earth before the mission (Earth: −62.8 ± 8.0 days), about 2 weeks after the launch (space1: 10.4 ± 1.9 days) and about 2 months after the launch (space2: 56.0 ± 3.4 days). Sleep quantity the night before the recording session was not different across conditions (Earth: 6.4 ± 0.4, space1: 7.0 ± 0.6, space2: 6.8 ± 0.4 h, linear mixed-effects model with Earth/space1/space2 as a fixed effect and different random intercepts for each astronaut, *F*(2,12) = 0.40, *p* = 0.679, *n* = 15 recording sessions) (Fig. 1a) and also the duration of wakefulness before the recording was not different across the three conditions: Earth (293 ± 45), space1 (327 ± 77) and space2 (367 ± 76 min) (linear mixed-effects model with Earth/space1/space2 as a fixed effect and different random intercepts for each astronaut, *F*(2,12) = 0.60, *p* = 0.564, *n* = 15 recording sessions) (Fig. 1b). Similarly, no significant difference was shown for the time of the recording and the wake up time (Supplementary Fig. 8). In the first step we assessed theta power’s topographical distribution across the scalp. Theta power showed a similar distribution across the three conditions (Fig. 2a). In the majority of astronauts we observed a global increase (i.e., when at least 50% of the electrodes are involved) in theta power from Earth to space (in five astronauts out of five for space1 and four out of five for space2). However, by comparing Earth to space no cluster of more than two electrodes showed a significant increase of theta power (df = 4, white dots uncorrected *p*-values < 0.05, *n* = 5 astronauts) (Fig. 2b) and a high variability can be observed across astronauts (Fig. 2c).

### Local sleep-like events’ amplitude and globality changes from Earth to space recordings

**Fig. 3:**
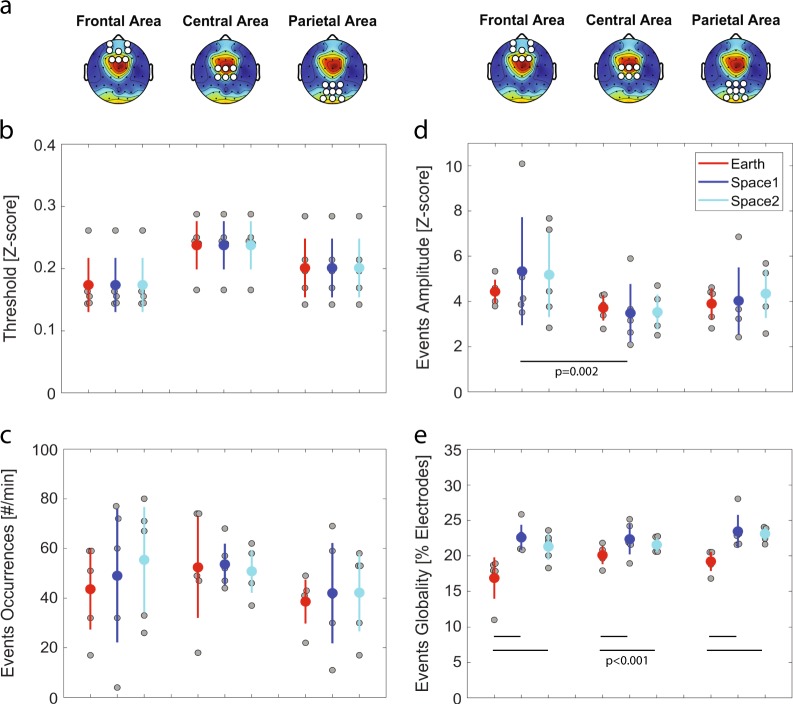
Local sleep-like events’ properties. **a** Topographical distribution of theta power in space2. The white dots define the three non-overlapping areas (frontal, central and parietal) used for the detection of local sleep-like events. **b** The voltage at each channel is standardised by *z*-score transform. A detection threshold is defined for each astronaut within each area (i.e., frontal, central or parietal area). Each grey dot represents the mean value for one astronaut and the mean ± sem across astronauts are colour marked as a dot and a line, respectively. **c** No difference in the number of local sleep-like events selected in each area and across conditions. **d** Increase of the amplitude for local sleep-like events in the frontal area compared to the central area. **e** Increase of the globality for local sleep-like events from Earth to space. (*n* = 5 astronauts)

To investigate sleep pressure markers during wakefulness, we detected local sleep-like events. (i) We first defined three areas of interest along the anterior-posterior axis (i.e., frontal, central and parietal)(Fig. 3a). (ii) Then, we defined specific detection thresholds according to theta oscillations within each of these areas (Fig. 3b). Specific thresholds were computed for each condition (i.e., Earth/space1/space2) and averaged within each astronaut. We show that the number of local sleep-like events did not vary by topographical areas (linear mixed-effects model with frontal/central/parietal as a fixed effect and different random intercepts for each astronaut, *F*(2,42) = 1.844, *p* = 0.171, *n* = 45 measures), nor by recording conditions (linear mixed-effects model with Earth/space1/space2 as a fixed effect and different random intercepts for each astronaut, *F*(2,42) = 0.28, *p* = 0.758, *n* = 45 measures) (Fig. 3c). (iii) To look further into the characteristics of local sleep-like events, we calculated the amplitude between the negative peak and the positive peak for each local sleep-like event (Fig. 3d). We also examined how many electrodes were involved in each event, defining this latest property as the globality of an event (Fig. 3e). Our results show that the amplitude varies across topographical areas (linear mixed-effects model with frontal/central/parietal as a fixed effect and different random intercepts for each astronaut, *F*(2,42) = 5.73, *p* = 0.006, *n* = 45 measures): The amplitude of the local sleep-like events is higher in the frontal area compared to the central area (*z*-score: +1.41 ± 0.42, *t* = 3.43, df = 42, *p* = 0.002). We also calculated the differences in globality of the detected events and we found that globality varies across conditions (linear mixed-effects model with Earth/space1/space2 as a fixed effect and different random intercepts for each astronaut, *F*(2,42) = 21.11, *p* < 0.001, *n* = 45 measures). Specifically, in space local sleep-like events are more global compared to Earth (Earth to space1: +4.06% ± 0.66, *t* = 6.13, df = 42, *p* < 0.001, Earth to space2: +3.26% ± 0.66, *t* = 4.92, df = 42, *p* < 0.001).

### Alertness and cognitive performances changes from Earth to space recordings

**Fig. 4:**
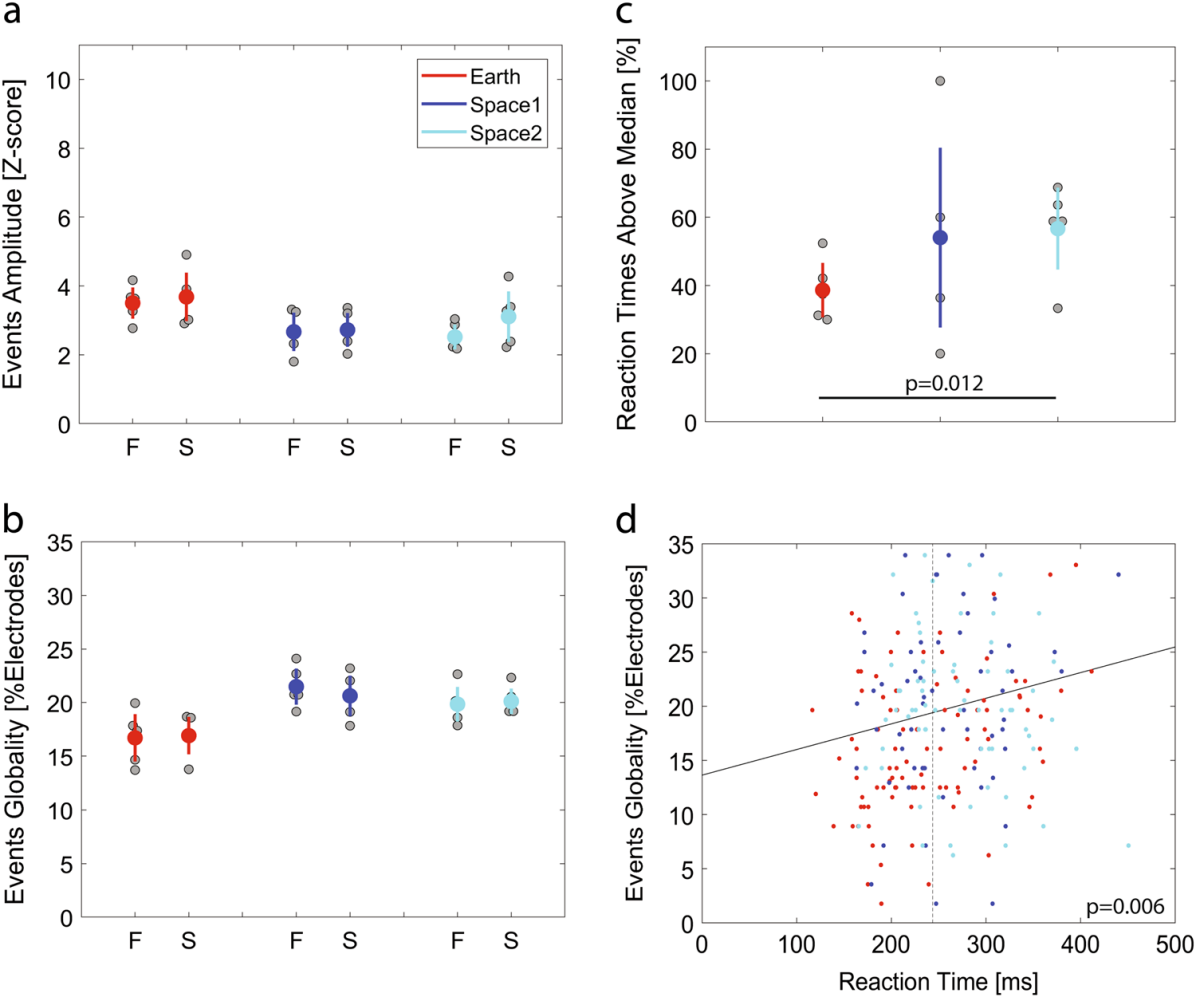
Local sleep-like events impact on cognitive tasks and alertness. **a**, **b** During the 10 s of the recovery manoeuvre, the astronauts either Failed (F) or Succeeded (S) to dock the simulated Soyuz vehicle. The two outcomes of the visuomotor task could not predict the local sleep-like events’ properties. Each grey dot represents the mean value for one astronaut and the mean ± sem across astronauts are colour marked as a dot and a line, respectively. **c** Reaction time until first movement while performing the recovery manoeuvre. There is a higher percentage of above median reaction times in space2 compared to Earth. **d** The full line illustrates the positive correlation between the local sleep-like events’ globality around the starting point of the recovery manoeuvre (−250 ms to 500 ms) and the reaction times across all trials. The median reaction time is marked by a dotted line at 244 ms. (*n* = 5 astronauts)

The visuomotor task performed by the astronauts for 25 min on the day of the experiment, which involves piloting and preparing the docking of a simulated Soyuz vehicle to the ISS, had two outcomes: (1) the astronauts succeeded in recovering and docking the simulated Soyuz vehicle or (2) they failed (Fig. 6). By looking into the local sleep-like events’ properties, the outcomes of the task could not be predicted based on the amplitude (generalised linear mixed-effects model, assuming a binomial response distribution, with events’ amplitude as a fixed effect and different random intercepts for each astronaut, *F*(1,453) = 0.01, *p* = 0.951, *n* = 455 trials), nor by the globality of the events (generalised linear mixed-effects model, assuming a binomial response distribution, with events’ globality as a fixed effect and different random intercepts for each astronaut, *F*(1,453) = 0.02, *p* = 0.893, *n* = 455 trials) (Fig. 4a, b). When carefully looking at reaction times, we observed a variation in the amount of reaction times above 244 ms (i.e., the median reaction time) from Earth to space (linear mixed-effects model with Earth/space1/space2 as a fixed effect, different random intercepts for each astronaut and by-astronaut random slopes for the fixed effect, *F*(2,12) = 4.52, *p* = 0.034, *n*_Earth_ = 5, *n*_space1_ = 3, *n*_space2_ = 4 astronauts) (Fig. 4c). Specifically, the percentage of reaction times above median (reaction time) increased by 18.03% ± 6.05 in space2 compared to Earth (*t* = 2.98, df = 12, *p* = 0.012). Moreover, looking at all reaction times across recording sessions, excluding outliers, it appears that local sleep-like events are more global during slower reaction times (linear regression model with events’ globality as a fixed effect: *R*^2^ = 0.03, *F* = 7.7, df = 235, *p* = 0.006, *n* = 237 trials) (Fig. 4d).

## Discussion

In this study, we observed a global increase of theta power in space compared to the Earth condition without any consistent topographical pattern. To further analyse sleep pressure markers and reveal potential negative effects of the ISS environment on sleep, we detected local sleep-like events. We showed that local sleep-like events are more widespread over the scalp (i.e., increased globality) in space compared to Earth. While analysing the outcomes of the visuomotor task, we observed an increase of above median reaction times after 2 months in space compared to Earth. By correlating sleep pressure markers with astronauts’ performances, we found a positive association between the globality of the local sleep-like events during the first movement and reaction times. However, local sleep-like events were not associated with the astronauts’ docking performances.

As expected from an electrophysiological marker of sleep pressure, numerous studies showed increased theta power after sleep deprivation.^14,16–18^ Thus, our observation that theta power was increased across the cortex in space compared to Earth might indicate increased sleep pressure in space. Studies using sleep deprivation also found that the increase in theta power was most pronounced over frontal midline areas.^14,18^ With only five astronauts, our study might have been under-powered to detect topographical changes in theta power. The individual topographical increase of power, however, was quite reproducible between Earth and space1 and Earth and space2, which indicates good signal quality across conditions. The effects of sleep restriction compared to sleep deprivation are typically less pronounced on the electrophysiological level.^26^ As the astronauts got about 6–7 h of sleep the night before the recordings, we would expect rather small changes in theta power, further limiting the likelihood to see local changes in theta power. However, our knowledge of the sleep-wake history is limited to the night before the recording.

Studies in rats and humans have shown that increased sleep pressure is associated with more widespread local sleep-like events.^21–24^ Thus, our observation that local sleep-like events are more widespread in space compared to Earth further supports increased sleep pressure in space. Increased sleep pressure is typically also associated with slower reaction times, a behavioural measure of a decrease in alertness.^27^ Although we found no change in general docking performance across the three conditions, we found a significant increase in above median reaction times in space. This observation is in agreement with the increased sleep pressure measured in space.

Interestingly, the studies investigating local sleep-like events have presented an association with task performance.^21–24^ Specifically, temporal proximity of task execution to more widespread local sleep-like event was associated with performance deterioration.^21,28^ We found a similar, though very weak, relationship in that globality of local sleep-like events was associated with slower reaction times. What compels a local sleep-like event to involve larger cortical areas is not fully understood. One explanation might be that more widespread local sleep-like events may represent an increased level of network synchronisation as a result of the homoeostatic build-up of learning related synaptic strength.^20^ Even though effects of increased sleep pressure on cognitive performances has been well established,^29,30^ direct implication of local sleep-like events’ globality on cognitive tasks would need to be further explored in a larger sample population.

Electrophysiological and behavioural markers indicate increased sleep pressure in space. Sleep pressure is regulated by two factors, a homoeostatic process and circadian rhythms. What underlays this increased sleep pressure in space is difficult to answer with our data. The homoeostatic build-up of sleep pressure is directly dependent on previous sleep and wake duration.^15^ Thus, we took great care in selecting recording sessions for which both the duration of sleep the night before, as well as the duration of wakefulness before the recording session were not significantly different across conditions. To do so we had to exclude a large number of preflight recordings, which means that in follow-up experiments, the time at which the astronauts are recorded should be better controlled. In the Neurospat experiment each astronaut was allocated 8.5 h for sleep the night before the recording and sleep quantity the night before the recording was not systematically different across the conditions. Accordingly, an increase of sleep pressure in space is likely not due to a loss of sleep quantity the night before the recording. However, experimental evidence using actigraphy data indicates that astronauts experience a lack of sleep quantity on Earth before the mission and on the ISS.^2,8,9^ In our study, we cannot exclude that astronauts were partially sleep restricted as we have no actigraphy recordings nor sleep diaries for the week before the recordings.

As introduced, sleep recovery is not only dependent on sleep quantity but also on sleep quality. Thus, our observation of increased sleep pressure could be due to a loss of sleep quality in space. To confirm a decrease of sleep quality we would need night EEG recordings. Unfortunately, such measurements were not available in the Neurospat experiment. Multiple factors could impact sleep quality in space: microgravity, confinement, chronic stress, temperature, light and noise disturbance.^3,4^ Unfortunately, the direct impact of each single space environmental factor on sleep quality could not be assessed within this study due to the limits of the experimental protocol. We have only two recording sessions in space and we are unable to show if our findings are impacted by microgravity per se (i.e., short-term effects). Thus, in microgravity, potential differences in the underlying mechanisms regulating local sleep-like events require further investigation.

Finally, circadian rhythms could also impact measures of sleep pressure.^15–17,31,32^ Indeed, astronauts have been shown to be often misaligned with their circadian clock during their mission.^8,9^ However, to assess such circadian alignment, we would need constant actigraphy recordings or sleep diaries along the mission. Unfortunately, none of these measurements were available for all the astronauts participating in the Neurospat experiment. To limit confounding effects of misalignment, the Neurospat experiments were not performed in the 48 h following air travel that involved a change of >4 time zones, nor following work shifts inducing >4 h of time shift, nor on the day after imposed sleep deprivation. Moreover, we were selective in choosing the included recording sessions. In regard to the time of day at which the recording took place, we made sure that they were not significantly different across the conditions. Nevertheless, as we do not have any information about circadian alignment the days before the recordings, we cannot fully exclude circadian misalignment.

With all the limitations in mind, our analysis provide initial evidence for increased sleep pressure in space. It would be interesting in future studies, involving more astronauts, to see whether these findings are related to task performance. Sleep quality might be an important factor to consider because it will be key for maintaining astronaut’s cognitive functions and improving missions’ success rate.

## Methods

### Participants and experiment

Five male astronauts (53 ± 1.6 years old) took part in the Neurospat experiment (AO-2004, 118). Written informed consent was obtained prior to participation. The experimental protocol was approved by the European Space Agency’s Medical Board (ESA-MB) and the NASA Johnson Space Centre Institutional Review Board (NASA-IRB). To ensure comparable levels of sleep quantity the night before the recordings, a sleep questionnaire was filled out by astronauts. Astronauts were allocated 8.5 h for sleep the night before the experiment and we excluded all recordings where astronauts reported sleeping <5 h. Neurospat experiments were not performed in the 48 h following air travel that involved a change of >4 time zones, nor following work shifts inducing >4 h of time shift, nor the day after imposed sleep deprivation, nor after a highly strenuous physical or mental activity such as extravehicular activities, centrifuge training, vestibular counter-measures experiments. Astronauts were instructed to maintain their normal consumption of caffeine but were not allowed alcohol nor medication 16 h before the experiment. Although Neurospat’s principal investigators asked to perform the experiments at the same time of day, preferably the morning, recordings took place at variable times along the day, between 2 and 10 h after awakening. In this study, we defined the recording conditions with the label Earth for the recordings on Earth and space1/space2 for the recordings on the ISS. During each Neurospat recording session, the astronauts had a resting state period, a visual orientation task and a visuomotor task to perform. Prior to Earth recordings, the astronauts had two training sessions on Earth to get familiar with the tasks. These two training sessions were not used in this analysis. Then, they had three recordings on Earth before the mission for each astronaut. Out of the three Earth recordings available in the Neurospat experiment, we included in our analysis only the Earth recording with a minimum difference in time after awakening compared to the space recordings. Finally the astronauts had two recordings on the ISS. We discarded post-flight recordings because they were recorded on another EEG system (Advanced Neuro Technology) at the Johnson Space Centre (Houston, USA).

### Wake EEG recordings

Each participant, for each session, was recorded with 58 EEG electrodes with the multi-electrode electroencephalogram mapping module (MEEMM) from the European physiology module placed on the ISS Columbus module, at the European Astronaut Centre (Köln, Germany) or at Star City (Moscow, Russia). In addition to the 58 EEG electrodes (10–20 electrode system EEG cap), three electrooculogram (EOG) (allowing horizontal and vertical EOGs), one electrocardiogram (ECG) and one electromyogram (EMG) (recorded at the first interosseous muscle of the right hand) were recorded. Continuous wake EEG was recorded for 70 min during each Neurospat session at a sampling rate of 1116 Hz (0.01–558 Hz band width). Scalp electrodes’ impedance were measured and kept below 5 KΩ. For all recordings, the reference was placed on the right earlobe. EOG, EMG, ECG and derivation P5 and P6 were excluded from further analysis. On Earth, the astronauts performed the experiment seated at a table. On the ISS Columbus module they were free floating with a secured loose-fitting leash around the waist and attached to the European Physiology Module rack.

### EEG pre-processing

EEG data pre-processing was performed in Matlab (Version R2017b) using EEGLAB toolbox scripts (Version 14)^33^ and additional custom made scripts. EEG data were pass-band filtered [0.1–48 Hz] and down sampled to 512 Hz. The signal was recorded as the difference of potential between the electrodes of interest and the right earlobe (i.e., earlobe referencing), which dampened the amplitudes of all oscillations close to the reference point and ultimately induced an asymmetry towards the left hemisphere. To correct for this effect, we transformed the data by subtracting the average activity across all electrodes (i.e., average referencing). A first Independent Component Analysis^34^ was performed to remove ocular, muscular, and electrocardiographic artefacts (Earth: 2.6 ± 0.7, space1: 1.0 ± 0.0, space2: 1.8 ± 0.5 components rejected) as defined by Hulse et al.^35^. Using the EEGLAB graphical user interface, all movement artefacts in the signal were marked by visual inspection and removed (Earth: 55.3 ± 2.2, space1: 60.6 ± 2.8, space2: 64.7 ± 4.4 min of recording remaining). The power spectrum was computed for each channel and outliers containing high muscle artefacts (20–30 Hz) were excluded from the dataset (Earth: 2.0 ± 0.7, space1: 1.6 ± 0.8, space2: 0.8 ± 0.3 channels rejected)^22^. A second independent component analysis was performed on cleaned data to further remove ocular, muscular, and electrocardiographic artefacts (Earth: 4.2 ± 0.9, space1: 4.0 ± 0.7, space2: 4.4 ± 0.8 components rejected). For each subject, rejected channels were interpolated. To the best of our knowledge, the prospect of an impedance difference at the electrodes between Earth and space has never been studied. With the assumption that such a difference could occur due to microgravity (e.g., electrical conduction differences), we prevented any effects of the recording montage on the signal by using a *z*-score transformation. Moreover, to improve the signal to noise ratio in the power spectrum analysis, we performed a phase-rectified signal averaging (PRSA).^36^ PRSA allows superimposing of the oscillations to create interference and hence reduce the weight of acute noise generators in the signal. The power spectral density was estimated using the Welch’s averaged periodograms with a four second Hamming window and a frequency resolution of 0.125 Hz. In each frequency bin, the power at each channel was normalised by the average power over the scalp. The theta power band was computed between 5 and 7 Hz, to stay distant from astronauts’ alpha peak (8–10 Hz).^37^ As in previous work,^23^ we define a global increase when >50% of the electrodes were involved.

### Local sleep-like events detection

**Fig. 5:**
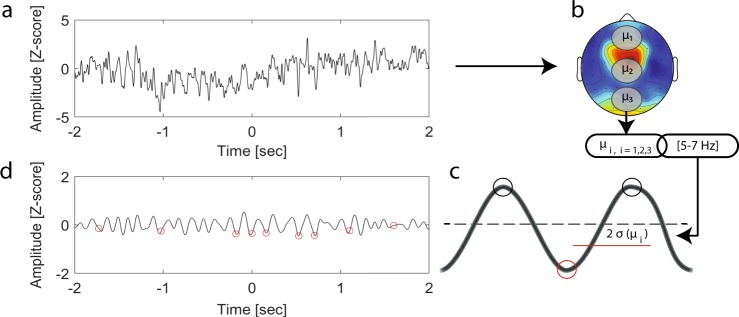
Local sleep-like event detection method. **a** Difference of potential at the derivation C3. **b** Signal average within three non-overlapping areas (frontal, central and parietal) and filtered within the theta band. **c** Detection threshold (red horizontal line) at two times the median deviation from the median signal within each area. All negative peaks on the reference signal below this relative threshold were detected and marked as a local sleep-like event. **d** Red circles mark the local sleep-like events involving the derivation C3

To look for evidence of local sleep-like events during wakefulness we combined existing detection methods.^22–24,38,39^ We used the SWA-Matlab toolbox developed by Mensen and colleagues with the following parameters^39,40^ (Fig. 5). The EEG channels within three non-overlapping areas (frontal electrodes: Fp1, Fp2, AF3, AFz, AF4, F1, Fz, F2, central electrodes: FC1, FCz, FC2, C1, CPz, CP2 and parietal electrodes: CP1, CPz, CP2, P1, Pz, P2, PO3, POz, PO4) were averaged and the three outcomes were filtered within the theta band using a second order Butterworth band pass filter. A threshold was set at two times the median deviation from the median signal for each area reference signal. To correct for potential remaining slow drifts in the EEG signals, we chose to detect local sleep-like events using the minimum negative point between two maximum peaks oscillating within theta oscillations, instead of the commonly used minimum negative point between two consecutive zero crossings. All negative peaks on the reference signal below this relative threshold were detected and marked as a local sleep-like event (7019 ± 488 events per recording session). To study the size of each local sleep-like event over the scalp, the event’s globality was computed by cross correlating the reference signal with each channel across the scalp within the theta range, looking for similar oscillations within a 50 ms time window. For each correlation above 95%, the corresponding channel was marked as involved in the event. As an additional layer of security, we unmarked isolated channels, which could represent artefacts by applying a cluster test.^41^ By defining a 50 ms time window to assess how many electrodes are involved in a local sleep-like event, we assumed that the theta waves are travelling over the scalp at least twice faster than slow waves.^22,38^ The number of areas of interest over the scalp defined the sensitivity of our detection algorithm. By choosing three areas of interest, we targeted only events within one of these areas and by averaging the signals within these areas, we targeted events involving at least a few electrodes. To refine the detection of events in specific cortical areas, we would need higher density EEG recordings, exceeding the current 58 electrodes. Moreover, average referencing will subtract the signal of the neighbouring electrodes, which might prevent our algorithm from detecting local sleep-like events involving only one electrode. Eventually, average referencing will induce a bias towards the detection of more global events compared to earlobe referencing. We further assessed the density of local sleep-like events per minute of recording and the amplitude from the negative peak to the following positive peak, measured at the channel with the median slope across all channels involved in the corresponding event.

### Visuomotor task

**Fig. 6:**
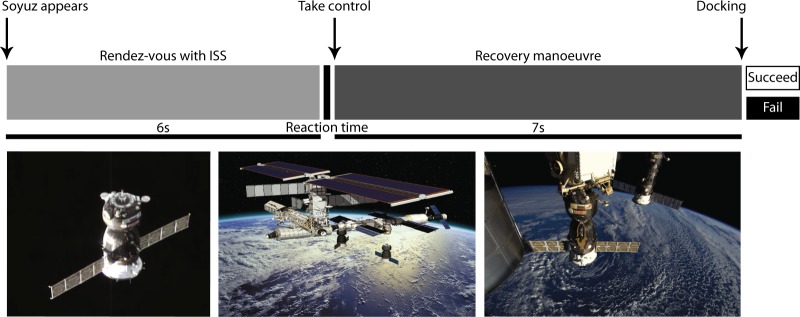
Visuomotor task experimental protocol. Rendez-vous with ISS (first 6 s) and recovery manoeuvre (following 7 s). The time when the astronauts are allowed to recover the simulated Soyuz vehicle until they take control of the spacecraft with their first movement is defined as reaction time. Photographs used from ESA, ESA-David Ducros and ESA/NASA with permission (photographs from left to right)

**Fig. 7:**
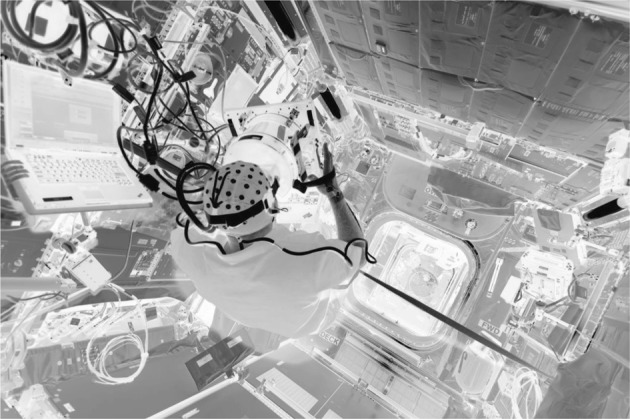
Astronaut performing the Neurospat experiment on the ISS Columbus module. Photograph used from ESA/NASA with permission

In the last part of the experimental protocol, the astronauts had to perform a visuomotor task that lasted  25 min (Fig. 6). The astronauts were told to look straight ahead at a laptop screen through a facemask to remove any external visual cues (Fig. 7). The astronauts were looking at a virtual display on a computer screen, simulating randomly two scenarios: one piloting the Soyuz vehicle, preparing a docking to the ISS (ISS being the target in this case) or the second while being within the ISS, preparing remotely the docking of the Soyuz vehicle (the Soyuz being the target in this second case). There were 80 trials per session (40 for each scenario). The 80 trials were divided in four blocks, which allowed the astronauts to take a break before starting the next block of 20 trials. At the beginning of each trial and for 2 s the astronauts saw first their own spaceship and then their target. Following this, the target deviated from its nominal straight-ahead position for another 2 s. Throughout this first period, the astronauts were asked to observe their target (ISS or Soyuz vehicle) without performing any movement. Six seconds after the beginning of the trial, the centre of the target changed from white to grey, which meant that the astronauts were required to take control of the spaceship and perform, as quickly as possible and in <7 s, the recovery manoeuvre towards the target by controlling a joystick with their right index finger. Once the docking position was reached, the astronauts were asked to confirm their attempt by pressing a button with their right thumb. The centre of the spaceship changed from white to blue if they successfully (S) docked the spaceship or to yellow if they failed (F). The next trial would start 2 s later. The astronauts had to perform the same experiment repeatedly during each recording session, bringing the total number of artefact free trials to 455 (Fail: 10.80 ± 1.00, Succeed: 21.27 ± 1.35 trials per session and per astronaut). The time point from which the astronauts were allowed to recover the trajectory of the spacecraft until the first movement recorded with the joystick was defined as reaction time. Only 15 reaction times were above 500 ms. They were considered as outliers and discarded from our analysis. Reaction times below 100 ms were due to anticipated movements (i.e., false start) and also discarded, reducing the total number of trials to 237.^27^ The time interval to look for local sleep-like events was defined as 250 ms before stimulus (i.e., motor action planning) and 500 ms after stimulus presentation (i.e., maximal reaction time).^42^

### Statistics

Data points were reported as mean ± sem. For topographical analysis we plotted *t*-values for the two-tailed paired Student’s *t*-tests (uncorrected *p*-values) and we used a non-parametric permutation test (coefficient of variation = 2.757, 2^5^ permutations, *n* = 5 astronauts) for cluster correction,^43,44^ defining the minimum cluster size of significant neighbouring electrodes for a pattern to be significant. In our analysis at least three neighbouring electrodes needed to be significant to be reported as a significant effect. For repeated measures time series, we used mixed-effects analysis to model repeated measures. First we assessed normal distribution with a quantile-quantile plot. For normal distributions, we performed a linear mixed-effects analysis of the relationship between the response variable and fixed/random effects. For non-normal distributions (e.g., binomial distributions), we used a generalised linear mixed-effects analysis. We used the restricted maximum likelihood estimate method to fit the model and choose the best model based on Bayesian information criterion results. Visual inspection by quantile-quantile plot of the residuals confirmed that homoscedasticity and normality were respected. The influence of the fixed effects on the model were determined by *F*-tests (*F*(degrees of freedom in the numerator, degrees of freedom in the denominator) = *F*-value, *p* = *p*-value). We finally reported the estimated differences (mean ± sem) between repeated measures for each fixed effect, together with the two-tailed paired Student’s *t*-tests results (*t* = *t*-values, df = degrees of freedom, *p* = *p*-values). When no fixed effects could help to fit the model (i.e., best model is the intercept only model), we reported the non-significant results for the *F*-test assessing the influence of each fixed effect candidate on the model. If the variable of interest is best modelled by a unique fixed effect without random effects, we used a linear regression model to obtain a *R*^*2*^ adjusted value, which indicates how much of the total variation can be explained by the fixed effect. Then, we completed a *F*-test (*F*-value, df = degrees of freedom, *p* = *p*-value) with the null hypothesis that the slope of the model is equal to zero. All statistical analysis were performed in Matlab.

### Reporting Summary

Further information on research design is available in the Nature Research Reporting Summary linked to this article.

## Supplementary information

Supplementary Figure 1

Reporting summary form

Supplementary Figure Legend

## Data Availability

All relevant data will be available from the corresponding authors upon request and after approval from the European Space Agency Medical Board (ESA-MB) and the NASA Johnson Space Centre Institutional Review Board (NASA-IRB).

## Supplementary information

**Supplementary information** accompanies the paper on the *npj Microgravity* website (10.1038/s41526-019-0069-0).

## Code availability

All relevant scripts will be available from the corresponding authors upon request.
