# Supplementary material for: Local sleep-like events during wakefulness and their relationship to decreased alertness in astronauts on ISS
Source: NPJ Microgravity. 2019 May 2;5:10. doi: 10.1038/s41526-019-0069-0 (PMC6497715; doi:10.1038/s41526-019-0069-0)
Supplement: Supplementary file 3 — Supplementary Figure Legend [file 41526_2019_69_MOESM3_ESM.docx]

Supplementary Figure 1: Three conditions: one on Earth, two in space. (a) For each

astronaut, the time of day when the recording was performed was not different across the three

conditions: Earth (12:3 _ 0:6), space1 (11:8 _ 1:6) and space2 (13:5 _ 1:6 hours) (linear mixedeffects

model with Earth/space1/space2 as a fixed effect and different random intercepts for each

astronaut, F(2,12)=0.56, p=0.583, n=15 recording sessions). (b) For each astronaut, the time

when they woke up on the day of the recording was not different across the three conditions:

Earth (7:2 _ 0:2), space1 (6:4 _ 0:5) and space2 (7:4 _ 0:7 hours) (linear mixed-effects model

with Earth/space1/space2 as a fixed effect and different random intercepts for each astronaut,

F(2,12)=0.98, p=0.405, n=15 recording sessions). (n=5 astronauts)
